# Mechanisms by which the infection of *Sclerotinia sclerotiorum (Lib.) de Bary* affects the photosynthetic performance in tobacco leaves

**DOI:** 10.1186/s12870-014-0240-4

**Published:** 2014-09-23

**Authors:** Cheng Yang, Zishan Zhang, Huiyuan Gao, Meijun Liu, Xingli Fan

**Affiliations:** State Key Lab of Crop Biology, College of Life Sciences, Shandong Agricultural University, Tai’an, Shandong 271018 China; Wheat Research Center, Henan Academy of Agricultural Sciences, Zhengzhou, Henan 450002 China

## Abstract

**Background:**

*Sclerotinia sclerotiorum (Lib.) de Bary* is a necrotrophic fungal pathogen which causes disease in a wide range of plants. An observed decrease in photosynthetic performance is the primary reason for the reduction of crop yield induced by *S. sclerotiorum*. The H_2_C_2_O_4_ is the main pathogenic material secreted by *S. sclerotiorum*, but the effects of H_2_C_2_O_4_ acidity and the C_2_O_4_^2−^ ion on photosynthetic performance remain unknown.

**Results:**

*S. sclerotiorum* infection significantly decreased photosynthetic O_2_ evolution and the maximum quantum yield of photosystem II (F_v_/F_m_) in tobacco leaves under high-light. H_2_C_2_O_4_ (the main pathogenic material secreted by *S. sclerotiorum*) with pH 4.0 also significantly decreased photosynthetic performance. However, treatment with H_3_PO_4_ and HCl at the same pH as H_2_C_2_O_4_ caused much less decrease in photosynthetic performance than H_2_C_2_O_4_ did. These results verify that the acidity of the H_2_C_2_O_4_ secreted by *S. sclerotiorum* was only partially responsible for the observed decreases in photosynthesis. Treatment with 40 mM K_2_C_2_O_4_ decreased F_v_/F_m_ by about 70% of the levels observed under 40 mM H_2_C_2_O_4_, which further demonstrates that C_2_O_4_^2−^ was the primary factor that impaired photosynthetic performance during *S. sclerotiorum* infection. K_2_C_2_O_4_ treatment did not further decrease photosynthetic performance when D1 protein synthesis was fully inhibited, indicating that C_2_O_4_^2−^ inhibited PSII by repressing D1 protein synthesis. It was observed that K_2_C_2_O_4_ treatment inhibited the rate of RuBP regeneration and carboxylation efficiency. In the presence of a carbon assimilation inhibitor, K_2_C_2_O_4_^2^ treatment did not further decrease photosynthetic performance, which infers that C_2_O_4_^2−^ inhibited PSII activity partly by repressing the carbon assimilation. In addition, it was showed that C_2_O_4_^2−^ treatment inhibited the PSII activity but not the PSI activity.

**Conclusions:**

This study demonstrated that the damage to the photosynthetic apparatus induced by *S. sclerotiorum* is not only caused by the acidity of H_2_C_2_O_4_, but also by C_2_O_4_^2−^ which plays a much more important role in damaging the photosynthetic apparatus. C_2_O_4_^2−^ inhibits PSII activity, as well as the rate of RuBP regeneration and carboxylation efficiency, leading to the over production of reactive oxygen species (ROS). By inhibiting the synthesis of D1, ROS may further accelerate PSII photoinhibition.

**Electronic supplementary material:**

The online version of this article (doi:10.1186/s12870-014-0240-4) contains supplementary material, which is available to authorized users.

## Background

*Sclerotinia sclerotiorum (Lib.) de Bary* is a necrotrophic fungal pathogen which causes disease in a wide range of plants, leading to enormous crop reduction [1,2]. Previous studies demonstrated that H_2_C_2_O_4_ is an important pathogenic determinant of *S. sclerotiorum. S. sclerotiorum* mutants deficient in oxalate biosynthesis were shown to be less pathogenic than wild-type fungus, and enhancement of H_2_C_2_O_4_ degradation capacity was shown to enhance plant resistance to *S. sclerotiorum* [3-5].

A great deal of researches have been conducted on the pathogenic mechanisms of *S. sclerotiorum*. It was reported that *S. sclerotiorum* can maintain maximal activity of cell wall-degrading enzymes such as polygalacturonase through the acid environment provided by H_2_C_2_O_4_ [6]. The cell wall is a natural barrier shield which protects plant tissues from pathogenic bacteria. *S. sclerotiorum* can weaken the cell wall through chelation of Ca^2+^ ions present in the cell wall with oxalic ions, breaking down Ca^2+^-dependent signal transduction pathways in the host plant [6]. NADPH oxidase, which is involved in reactive oxygen species generation, is required for the pathogenic development and is important for ROS regulation in the successful pathogenesis of *S. sclerotiorum* [7]. Recently, it was also reported that *S. sclerotiorum* (via H_2_C_2_O_4_) generates reducing conditions that suppress host defense responses, including the oxidative burst and callose deposition, during the initial stages of infection. However, once infection is established, *S. sclerotiorum* benefits to its infection by inducing the generation of plant ROS and programmed cell death (PCD) [7,8].

The leaves of host plant, the primary sites of photosynthesis, are the main targets of many pathogens. The infection of pathogens to the leaves directly reduces photosynthetic performance, leading to drastic losses in crop yield. The repression of photosynthesis in host plants induced by pathogens has been reported in many plant species. For example, photosynthesis was decreased in barley infected by *B. graminis* [9]. And down-regulation of photosystem II quantum yield in host plants occurred during the infection of *Pseudomonas syringae* [10], *Albugo candida* [11], *Puccinia coronata* and *Blumeria graminis* [9,12], as well as *Botrytis cinerea* [13]. It was also reported that the expression of sugar-regulated photosynthetic genes, such as the small subunit of ribulose-1,5-bisphosphate carboxylase and chlorophyll *a*, *b* binding protein, in most cases, were down-regulated after pathogen infection [14].

*S. sclerotiorum* infection could also decrease photosynthetic performance in host plants. During *S. sclerotiorum* infection, H_2_C_2_O_4_ promotes the accumulation of osmotically active molecules, inducing stomatal opening and inhibiting ABA induced stomatal closure, leading to foliar wilting [15]. In our previous work, *S. sclerotiorum* infection was shown to induce the over-accumulation of H_2_O_2_ in cucumber leaves due to inhibition of the activity of catalase, damaging the functions of photosystem I (PSI) and photosystem II (PSII) [16]. Although H_2_C_2_O_4_ was determined to be the main toxin secreted by *S. sclerotiorum*, it is still unknown whether the destructive effect of *S. sclerotiorum* on the host photosynthetic apparatus is due to the acidity of H_2_C_2_O_4_ or the effect of the C_2_O_4_^2−^ anion, and if either of them has a deleterious effect, what is the mechanism of impairment of photosynthetic performance?

In order to address this question, we compared the effect of H_2_C_2_O_4_ acidity and C_2_O_4_^2−^ ion on the photosynthetic performance of tobacco leaves, and studied the effects of C_2_O_4_^2−^ on the photosynthetic electron transport chain, carbon assimilation, the production of ROS and the synthesis of D1 protein in tobacco leaves under high-light treatment. The PSI activity and cyclic electron transport activity were also measured using modulated 820 nm reflection (MR_820 nm_) techniques [17-19].

## Results

### Effect of *S. sclerotiorum* infection on O_2_ evolution and PSII activity of leaves

**Figure 1:**
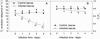
**Changes in the photosynthetic O**_**2**_ **evolution rate (A) and the maximum quantum yield of primary photochemistry (F**_**v**_ **/F**_**m**_ **, B) in tobacco leaves infected with** ***S. sclerotiorum*** **.** ●, ○ indicate control and infected leaves, respectively. Control leaves were leaves without infection by *S. sclerotiorum*. Leaf discs used for O_2_ evolution rate determination and F_v_/F_m_ measurement were cut within the region 2 cm away from the centre of the necrotic spot with a 10-mm cork borer. Different letters indicate significant differences between the leaves with different treatments (P < 0.05). Values were means ± SE (n = 8).

The O_2_ evolution rate reflects the capacity of the photosynthetic apparatus, including both the electron transport chain and carbon assimilation. The fluorescence parameter F_v_/F_m_, providing an estimate of the maximum quantum yield of primary photochemistry, is widely used to reflect the extent of photoinhibition [20,21]. Both the photosynthetic O_2_ evolution rate and F_v_/F_m_ decreased significantly in leaves infected with *S. sclerotiorum* when compared with controls, and the decreasing extent increased with the lasting of infection (Figure 1). This result indicates that *S. sclerotiorum* infection significantly inhibited photosynthesis and aggravated the photoinhibition in tobacco leaves under high-light.

### The effect of H_2_C_2_O_4_ and other acids on the activity of PSII

It has been proved that H_2_C_2_O_4_ is the primary pathogenic material secreted by *S. sclerotiorum* [22-24]. Additionally, injection of exogenous H_2_C_2_O_4_ can mimic disease symptoms of an actual fungal infection [6,25]. So H_2_C_2_O_4_ was used in the following experiments to study the effect of *S. sclerotiorum* on the photosynthetic performance in host plants. Because the pH and concentration of H_2_C_2_O_4_ in the culture solution of *S. sclerotiorum* were 4.0 and 40 mM respectively after 14 days growing of the *S. sclerotiorum*, 40 mM H_2_C_2_O_4_ with pH 4.0 (adjusted with KOH) was used to treat leaves in this experiment.

In order to distinguish the effect of H_2_C_2_O_4_-mediated acidity from the effect of C_2_O_4_^2−^ anion on photosynthetic performance, the effects of acidity (pH 4.0) provided by H_2_C_2_O_4_ (40 mM, pH adjusted to 4.0 with KOH), H_3_PO_4_ and HCl, respectively, were compared to 40 mM K_2_C_2_O_4_.

**Figure 2:**
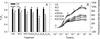
**Effect of HCl (pH 4.0), H**_**3**_ **PO**_**4**_ **(pH 4.0), H**_**2**_ **C**_**2**_ **O**_**4**_ **(40 mM, pH adjusted to 4.0), K**_**2**_ **C**_**2**_ **O**_**4**_ **(40 mM) and KCl (80 mM) treatment on F**_**v**_ **/F**_**m**_ **(A) and OJIP curves (normalized, B) in tobacco leaves.** ■ indicates leaves before treatment; □ indicates leaves 2 hours after high-light treatment. Leaf discs (10 mm diameter) were infiltrated with HCL (pH 4.0), H_3_PO_4_ (pH 4.0), H_2_C_2_O_4_ (40 mM, pH adjusted to 4.0), K_2_C_2_O_4_ (40 mM) and KCl (80 mM) under darkness for 3 h, followed by exposure to high-light (800 μmol m^−2^ s^−1^) for 2 hours. CK were leaves without any reagent treatment. Different letters indicate significant differences between leaves with different treatments (P < 0.05). Values were means ± SE (n = 8).

The F_v_/F_m_ decreased significantly in leaves treated with HCl (pH 4.0), H_3_PO_4_ (pH 4.0), H_2_C_2_O_4_ (40 mM, pH adjusted to 4.0) and K_2_C_2_O_4_ (40 mM) after high-light treatment (Figure 2A). The decrease of F_v_/F_m_ in HCl and H_3_PO_4_ treated leaves was only slightly lower than that in CK leaves after high-light treatment. However, H_2_C_2_O_4_ treatment significantly decreased F_v_/F_m_ in tobacco leaves (Figure 2). When K_2_C_2_O_4_ was used to eliminate H_2_C_2_O_4_ acidity in the experiment, the PSII activity was also severely inhibited under high-light. The extent of K_2_C_2_O_4_ inhibition of F_v_/F_m_ reached 69.7% of the inhibition induced by H_2_C_2_O_4_ (Figure 2A). Meanwhile, the F_m_ of the normalized fluorescence transient in K_2_C_2_O_4_ treated leaves decreased much more than that in HCl and H_3_PO_4_ treated leaves (Figure 2B), and the decreased extent was only a bit smaller than that in H_2_C_2_O_4_ treated leaves. The results indicate that the influence of H_2_C_2_O_4_ on photosynthetic performance was mainly caused by the C_2_O_4_^2−^ anion.

### The effect of different concentrations of K_2_C_2_O_4_ on the activity of PSII

**Figure 3:**
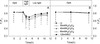
**Effect of different concentrations of K**_**2**_ **C**_**2**_ **O**_**4**_ **and KCl on F**_**v**_ **/F**_**m**_ **(A) and OJIP curves (normalized, B) in tobacco leaves.** In **A**, leaves were kept in the dark from 0–3 h, 800 μmol m^−2^ s^−1^ light from 3–5 h, and 50 μmol · m^−2^ · s^−1^ light from 5–11 h to recover. In **B**, the leaves were kept in the dark the duration of the study. CK were leaves without any reagent treatment. Different letters indicate significant differences between leaves with different treatments (P < 0.05). Values were means ± SE (n = 8).

In leaves treated with 20, 40, 60 mM K_2_C_2_O_4_ and 120 mM KCl, F_v_/F_m_ decreased significantly in high light (800 μmol · cm^−2^ · s^−1^; Figure 3A). When the treated leaves were placed in low light (50 μmol · cm^−2^ · s^−1^) for recovery after photoinhibition treatment, the F_v_/F_m_ in all leaves recovered to a large extent. However, the F_v_/F_m_ in K_2_C_2_O_4_ treated leaves recovered less than that in the control and KCl treated leaves (Figure 3A). Nonetheless, no significant differences were observed in F_v_/F_m_ between different treatment groups in the dark (Figure 3B). There was no significant difference in F_v_/F_m_ between KCl treated leaves and the control leaves either. Because 120 mM KCl has the same concentration of K^+^ as 60 mM K_2_C_2_O_4_ does, and it has lower osmotic potential than 60 mM K_2_C_2_O_4_, the effect of K^+^ and osmotic stress on the result was then neglected.

**Figure 4:**
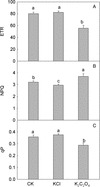
**Effect of K**_**2**_ **C**_**2**_ **O**_**4**_ **(40 mM) and KCl (80 mM) treatment on ETR (A), NPQ (B) and qP (C) in tobacco leaves.** Leaf discs (10 mm diameter) were infiltrated with K_2_C_2_O_4_ (40 mM) and KCl (80 mM) under darkness for 3 h, followed by exposure to intense light (800 μmol m^−2^ s^−1^) for 2 hours. CK were leaves without any reagent treatment. Different letters indicate significant differences between leaves with different treatments (P < 0.05). Values were means ± SE (n = 8).

In high light (800 μmol · cm^−2^ · s^−1^) for 2 hours, the electron transport rate (ETR) and photochemical quenching (qP) in K_2_C_2_O_4_ treated leaves decreased much more than that in control and KCl treated leaves, meanwhile, NPQ was higher in K_2_C_2_O_4_ treated leaves than that in the leaves with other treatment (Figure 4). This result further indicates that K_2_C_2_O_4_ treatment aggravates PSII photoinhibition in tobacco leaves under high-light.

**Figure 5:**
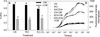
**The effect of 40 mM K**_**2**_ **C**_**2**_ **O**_**4**_ **and 80 mM KCl treatment on the F**_**v**_ **/F**_**m**_ **(A) and OJIP curves (normalized, B) in tobacco leaves in the presence of chloramphenicol (CM).** ■, − CM; □, + CM. Leaf discs (10 mm diameter) were immersed into solutions with 1 mM CM for 3 h in the dark, then kept in high light (800 μmol m^−2^ s^−1^) for 2 h. F_v_/F_m_ and Ψ_o_ were measured after 2 h high light treatment. CK were leaves without K_2_C_2_O_4_ and KCl treatment. Different letters indicate significant differences between leaves with different treatments (P < 0.05). Values were means ± SE (n = 8).

In the presence of chloramphenicol (CM), an inhibitor of de novo D1 protein synthesis [26,27], K_2_C_2_O_4_ treatment did not aggravate the decrease of F_v_/F_m_ (Figure 5). And the large difference in P point of the OJIP curves between different treatments was eliminated by CM treatment, which indicates that the inhibition of D1 protein by C_2_O_4_^2−^ accelerated the photoinhibition of PSII in leaves treated with H_2_C_2_O_4_ under high-light.

### The effect of K_2_C_2_O_4_ treatment on the accumulation of hydrogen peroxide (H_2_O_2_)

**Figure 6:**
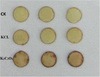
**The accumulation of H**_**2**_ **O**_**2**_ **in tobacco leaves after treatment with H**_**2**_ **O, 80 mM KCl and 40 mM K**_**2**_ **C**_**2**_ **O**_**4**_ **under high light (800 μmol m** ^**−2**^ **s** ^**−1**^ **) for 2 hours.** Histochemical detection of H_2_O_2_ production with 1 mg/ml 3, 3-diaminobenzidine (DAB, pH 5.5) staining. CK were leaves without K_2_C_2_O_4_ and KCl treatment. Representative images from five independent experiments are shown in the figure.

As shown in Figure 6, K_2_C_2_O_4_ treatment obviously enhanced the accumulation of H_2_O_2_ in tobacco leaves under high light when compared with control and KCl treated leaves, which indicates that K_2_C_2_O_4_ treated leaves suffered from greater photo-oxidative stress.

### The effect of K_2_C_2_O_4_ on carbon assimilation

**Figure 7:**
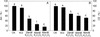
**Relative photosynthetic rates at saturated CO**_**2**_ **(Am, A), and carboxylation efficiency (CE, B) of tobacco leaves treated with different concentrations (0, 20, 40, 60 mM) of K**_**2**_ **C**_**2**_ **O**_**4**_ **and 120 mM KCl.** The petioles of detached leaves were dipped into treatment solutions before measurement in the dark for 3 hours. CK were leaves without K_2_C_2_O_4_ and KCl treatment. Different letters indicate significant differences between leaves with different treatments (P < 0.05). Values shown are means ± SE (n = 6).

**Figure 8:**
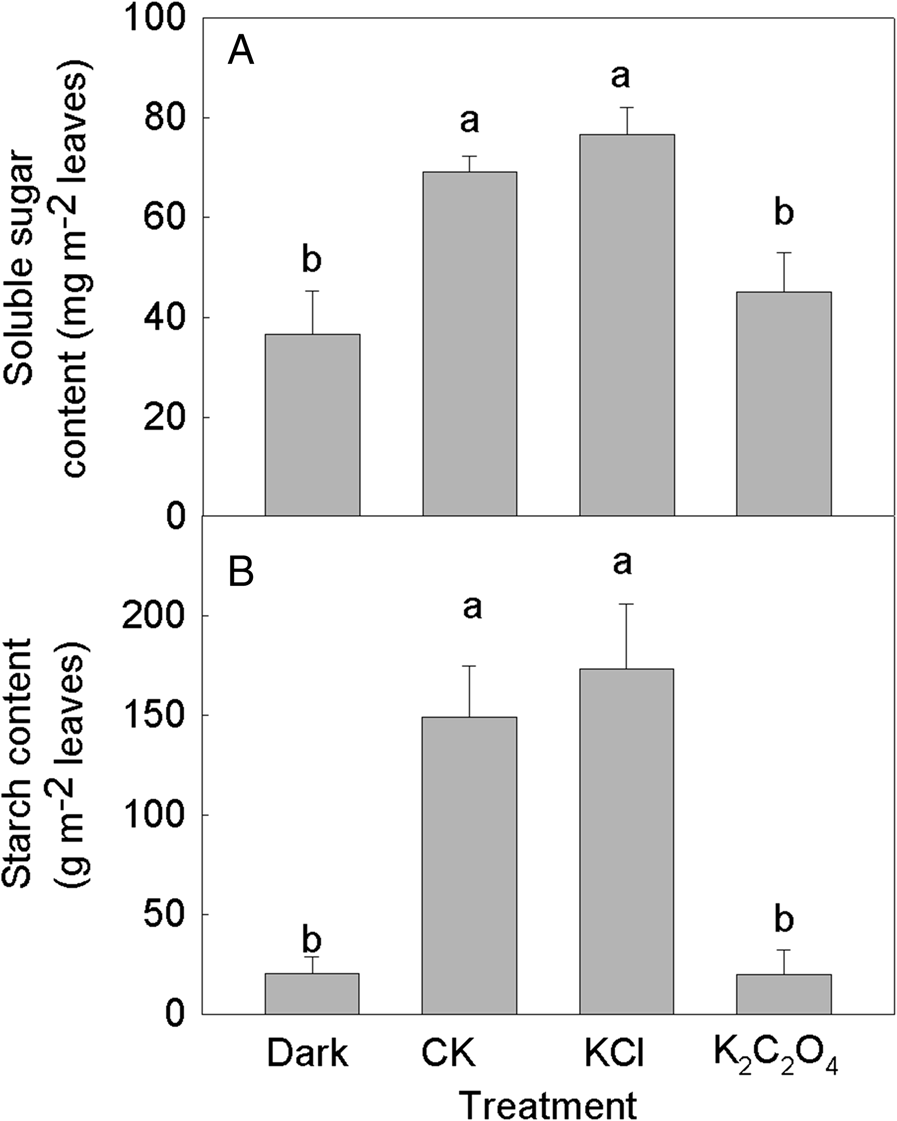
**The effect of K**_**2**_ **C**_**2**_ **O**_**4**_ **and KCl treatment on the content of soluble sugar (A) and starch (B) in tobacco leaves under light (800 μmol m** ^**−2**^ **s** ^**−1**^ **) for 2 hours.** The “Dark” were dark adapted leaves without any reagent treatment. Different letters indicate significant differences between leaves with different treatments (P < 0.05). Values shown are means ± SE (n = 5).

A photosynthetic model suggests that CO_2_ assimilation in C3 plants is limited by the rate of RuBP regeneration at high levels of CO_2_, and is limited by the efficiency of Rubisco at lower levels of CO_2_ [28]. In tobacco leaves treated with K_2_C_2_O_4_, carboxylation efficiency (CE) and net photosynthetic rate (Pn) at saturated CO_2_ (Am) both decreased severely compared to the control, at magnitudes which increased with K_2_C_2_O_4_ concentration (Figure 7). However, no significant decreases in CE and Am were observed in leaves treated with 120 mM KCl. It suggests that C_2_O_4_^2−^ inhibited both Rubisco activity and RuBP regeneration. In addition, after exposed to light for 2 h, the contents of both soluble sugar and starch in K_2_C_2_O_4_ treated leaves were significantly lower than those in control and KCl treated leaves (Figure 8), which demonstrates that C_2_O_4_^2−^ significantly inhibited Calvin cycle.

**Figure 9:**
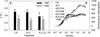
**The effect of 40 mM K**_**2**_ **C**_**2**_ **O**_**4**_ **and 80 mM KCl treatment on the F**_**v**_ **/F**_**m**_ **(A) and OJIP curves (normalized, B) in tobacco leaves under light (800 μmol m** ^**−2**^ **s** ^**−1**^ **) with or without 1 mM iodoacetamide (IAM).** CK were leaves without K_2_C_2_O_4_ and KCl treatment. Different letters indicate significant differences between leaves with different treatments (P < 0.05). Values shown are means ± SE (n = 8).

To further investigate the effect of K_2_C_2_O_4_ on carbon assimilation, leaves were pretreated with iodoacetamide (IAM), an inhibitor of the Calvin cycle [29,30]. In the presence of IAM, no significant differences were observed in F_v_/F_m_ between K_2_C_2_O_4_ treated leaves and control leaves after high light treatment (Figure 9). This result implies that when CO_2_ assimilation was inhibited, K_2_C_2_O_4_ didn’t further aggravate the photoinhibition under high light proving that the severe photoinhibition caused by the K_2_C_2_O_4_ may be due in part to inhibition of the Calvin cycle. The inference was also supported by the fact that IAM treatment eliminated the difference in OJIP curves between K_2_C_2_O_4_ treated leaves and CK.

### The effect of K_2_C_2_O_4_ on PSI activity and cyclic electron flow

**Figure 10:**
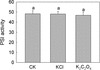
**The effect of K**_**2**_ **C**_**2**_ **O**_**4**_ **and KCl treatment on the PSI activity.** The PSI activity of the treated leaves were measured with an M-PEA (Hansatech Instrument *Ltd*., UK). The induction curve of MR_820 nm_ of the leaves obtained by saturating red light showed a fast oxidation phase and a following reduction phase. The initial slope of the oxidation phase of MR_820nm_ at the beginning of the saturated red light indicates the capability of P700 to get oxidized, which is used to reflect the activity of PSI.

**Figure 11:**
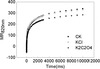
**The effect of K**_**2**_ **C**_**2**_ **O**_**4**_ **and KCl treatment on the cyclic electron flow.** The treated leaves were illuminated with a 20 s far-red light, then turn off the light and record the changes of the MR_820nm_ signal. The initial rate of MR_820nm_ indicates the activity of cyclic electron flow.

The PSI activities in control leaves, and leaves treated with KCl and K_2_C_2_O_4_ were measured. No significant differences were observed in PSI activity between leaves with different treatments (Figure 10). However, the initial increase rate of MR_820nm_ in K_2_C_2_O_4_ treated leaves after far-red illumination significantly decreased (Figure 11), which indicates a decrease in cyclic electron flow in K_2_C_2_O_4_ treated leaves.

## Discussion

*S. sclerotiorum* infection significantly inhibited photosynthesis (Figure 1), which suggests that the photosynthetic apparatus is a major pathogenic target. In this study, we demonstrated that H_2_C_2_O_4_, the most important pathogenic determinant of *S. sclerotiorum* infection, inhibited photosynthetic activity in tobacco leaves more severely than HCl and H_3_PO_4_ did at the same pH. The C_2_O_4_^2−^ anion appeared to play a more important role in damaging the photosynthetic performance than the acidity did, which was supported by the fact that the inhibition of K_2_C_2_O_4_ on PSII activity reached about 70% of the effect observed with H_2_C_2_O_4_ (Figure 2).

Therefore, K_2_C_2_O_4_ was used to study the mechanisms by which *S. sclerotiorum* affects photosynthetic performance in tobacco leaves. It has been known that the infection of *S. sclerotiorum* decreases chlorophyll content and causes the appearance of chlorotic symptom in host plants. However, the decrease in chlorophyll content often occurs after infection for several days [31,32], in our experiment, the treatment period is only about 5 h, so the decrease in chlorophyll content didn’t occur in the experiment (Additional file 1: Figure S1). The result indicates that the effect of K_2_C_2_O_4_ on photosynthetic apparatus does not depend on the degradation of chlorophyll.

Fv/Fm, ETR and qP decreased significantly in K_2_C_2_O_4_ treated leaves but the NPQ increased, indicating that the activity of PSII was significantly damaged by C_2_O_4_^2−^.We propose that during *S. sclerotiorum* infection, the decreased PSII activity was probably due to the damage to the reaction centre and acceptor side of PSII by C_2_O_4_^2−^ secreted by *S. sclerotiorum* because there was no difference observed in W_k_ (an indicator of the activity of the donor side of PSII [33,34]) between K_2_C_2_O_4_ treated leaves and control leaves (Additional file 2: Figure S2), and Ψ_o_ (an indicator of electron transport at PSII acceptor side [35]) decreased in K_2_C_2_O_4_ treated leaves (Additional file 3: Figure S3). In the dark, no significant difference was observed in F_v_/F_m_ between K_2_C_2_O_4_ treated leaves and control (Figure 4), demonstrating that C_2_O_4_^2−^ inhibited the photosynthetic electron transfer chain in an indirect, light-dependent manner.

PSII photoinhibition occurs when the absorbed light is more than it can be used by photochemistry [36,37]. However, photosynthetic organisms are able to overcome PSII photoinhibition by rapidly and efficiently repairing the damage, which requires the synthesis of proteins de novo, such as D1 [26]. Under high-light, the PSII activity depends on the balance between the rates of photodamage and repair; consequently, photoinhibition of PSII becomes apparent when the rate of photodamage exceeds the rate of repair [38-40]. As observed in the study, in the presence of CM, the K_2_C_2_O_4_-mediated decrease of F_v_/F_m_ and changes of OJIP curves were eliminated (Figure 5), which indicates that inactivation of PSII by the C_2_O_4_^2−^ was largely caused by inhibition of D1 protein synthesis. Recently, it was suggested that all environmental stresses enhance photoinhibition through an indirect method - promoting the production of ROS to inhibit the repair of the D1 protein [40,41]. In this study, a marked increase of H_2_O_2_ was observed in leaves treated with C_2_O_4_^2−^ (Figure 6), inferring that the increase of the ROS may inhibit D1 protein synthesis. However, further study is needed to clarify whether D1 protein synthesis was inhibited by accumulation of ROS caused by C_2_O_4_^2−^, or via some combination of the accumulation of ROS and C_2_O_4_^2−^ direct effect.

The chloroplast photosynthetic electron transport chain is one of the major sites of ROS production in leaves of green plants [42-44]. The photosynthetic electron transport and carbon assimilation work coordinately under normal physiological conditions. The damage or inhibition of either one of the two components would lead to increased ROS production. The fact that both the CE and Am were significantly inhibited by C_2_O_4_^2−^ indicates that carbon assimilation processes, including both Rubisco activity and RuBP regeneration (Figure 7 and Additional file 4: Figure S4), were inhibited by C_2_O_4_^2−^ under high-light, which is further supported by the decrease in the content of soluble sugar and starch in K_2_C_2_O_4_ treated leaves (Figure 8). We suggest that the over accumulation of ROS resulted from the inhibition of CO_2_ assimilation enhanced C_2_O_4_^2−^ mediated photoinhibition. This was further supported by the fact that IAM treatment eliminated the decrease in Fv/Fm (Figure 9) and the changes of OJIP curves induced by K_2_C_2_O_4_ under high-light.

It is known that C_2_O_4_^2−^ can react with many metal ions to form chelate compounds. Rubisco and fructose-1, 6-bisphosphatase are key enzymes in Calvin cycle. It has been reported that Ca^2+^ and Mg^2+^ play important roles in the regulation of the activity of chloroplast fructose-1,6-bisphosphatase [45,46], and Mg^2+^ has a enhancing effect on the Rubisco activity [47]. We presume the increased C_2_O_4_^2−^ in plant cells may decrease the activity of Rubisco or fructose-1,6-bisphosphatase through forming chelate compounds with Ca^2+^ or Mg^2+^ in chloroplast. Moreover, the signal role of H_2_C_2_O_4_ during the infection of *S. sclerotiorum* has been demonstrated [7,8,48], the increased C_2_O_4_^2−^ in plant cells may also interfere with CO_2_ assimilation through signal transduction. However, to determine the specific mechanism by which C_2_O_4_^2−^ inhibit the Calvin cycle needs further studies.

Under high-light treatment, though PSI activity was not damaged by C_2_O_4_^2−^ (Figure 10), cyclic electron flow around PSI was decreased by K_2_C_2_O_4_ treatment (Figure 11). The over-reducing of PSI acceptors, always leading to the increase of the generation of superoxide, would be prevented by an increase of cyclic electron flow around PSI [49]. Moreover, the cyclic electron flow can also decrease the probability that singlet oxygen generates within PSII through charge recombination [50,51]. Further studies are needed to clarify whether the decrease in cyclic electron flow induced by C_2_O_4_^2−^ is correlated to the increase in ROS generation and the enhancement of PSII photoinhibition in K_2_C_2_O_4_ treated leaves.

Our experiment demonstrated that it is the C_2_O_4_^2−^ ion secreted by *S. sclerotiorum* rather than the decrease in pH caused by the H_2_C_2_O_4_ that mainly induces the damage to photosynthetic apparatus. However, we did not exclude that necrosis may also cause inhibition of Calvin cycle and PSII activity. Since the C_2_O_4_^2−^ ion plays a more important role in impairing photosynthetic apparatus than acidity does, it reasonable to infer that the inhibition of Calvin cycle and PSII activity by necrosis in *S. sclerotiorum* infected leaves is mainly induced by the C_2_O_4_^2−^ ion secreted by *S. sclerotiorum*. F_o_ is the fluorescence emitted by antenna pigment of PSII with open reaction center. Chlorophyll content and F_o_ all showed no significant difference among the leaves treated with K_2_C_2_O_4_, CK and KCl, which indicates that the antenna of PSII might not affected by C_2_O_4_^2−^ in our experiment (Additional file 1: Figure S1 and Additional file 5: Figure S5).

## Conclusions

This study demonstrated that H_2_C_2_O_4_ secreted by *S. sclerotiorum* enhanced photoinhibition, mainly by the effect of the C_2_O_4_^2−^ ion. C_2_O_4_^2−^ led to the decrease of both the activity of Rubisco and RuBP regeneration, leading to the accumulation of H_2_O_2_ in the chloroplast. The over accumulation of H_2_O_2_ inhibited the turnover of the D1 protein. This is likely the primary mechanism by which *S. Sclerotiorum* infection affects the photosynthetic performance of tobacco leaves. Further studies are needed to explore whether C_2_O_4_^2−^ has a direct effect on D1 protein synthesis, and to elucidate the detailed mechanisms by which C_2_O_4_^2−^ inhibits the activity of Rubisco and RuBP regeneration in tobacco leaves. And if the inhibition of Rubisco activity and RuBP regeneration is mediated by the signal effect of C_2_O_4_^2−^ and if the decrease in PSII activity caused by C_2_O_4_^2−^ is involved in the PCD induced by *S.Sclerotiorum* remain to be elucidated in future work.

## Methods

### Plant materials

Tobacco seeds (Nicotiana tabacum L. cv. NC89) were germinated on vermiculite. Thirty days after germination, the seedlings were transplanted to pots containing a compost soil substrate to grow in a greenhouse under a natural photoperiod (day: 25–30°C, night: 20–25°C). Commercial humus, vermiculite and field soil (1:1:1, v:v:v) were mixed as a compost soil substrate. The pots were periodically irrigated with tap water and fertilized twice a month. Just before flowering, the new fully expanded leaves were used in this experiment.

### Fungal growth and plant inoculations

*S. sclerotiorum* was grown on PDA culture at 25°C in the dark for 3–5 days. After this period, mycelial agar plugs of 10 mm diameter were excised and transferred to Maxwell & Lumsden liquid cultures at 25°C in the dark for 14 days. The resultant mycelium was taken for use in leaf inoculation, which was performed according to Walz (2008) and Bu (2009) [16,52]. Leaf segments 2 cm away from the center of the necrotic spot were cut for measurement of O_2_ evolution rate and F_v_/F_m_.

### Photosynthetic O_2_ evolution rate measurement

A Chlorolab-2 liquid-phase oxygen electrode system (Hansatech Instruments, Norfolk, UK) was used to measure the photosynthetic O_2_ evolution rates of infected leaf discs in 1 mM NaHCO_3_ solution under saturation light (800 μmol m^−2^ s^−1^) at room temperature.

### Treatment of plant materials

Leaf disks (10 mm diameter) obtained from new fully expanded leaves were immersed in H_3_PO_4_ (pH 4.0), HCL (pH 4.0), H_2_C_2_O_4_ (40 mM, pH adjusted to 4.0 with KOH), 40 mM K_2_C_2_O_4_, or different concentrations (20, 40, 60 mM) of K_2_C_2_O_4_ solution for 3 hours in the dark for sufficient permeation, and then floated on the solution for photoinhibition or recovery treatment. *S. sclerotiorum* culture medium showed pH ~4.0 and [H_2_C_2_O_4_] ~40 mM; H_3_PO_4_, HCL were tested at the same pH. The photoinhibition and recovery of the leaf disks were taken under 800 μmol · m^−2^ · s^−1^ light and 50 μmol · m^−2^ · s^−1^ light, respectively.

The first new fully expanded leaves from the top of the tobacco plants were excised from the plants at the end of the petiole. The petioles of the excised leaves were quickly dipped into treatment solutions with a second excision in the solution. Treated leaves were then transferred into a growth chamber at 25°C in the dark. Gas exchange parameters were measured after four hours.

### Measurement of chlorophyll fluorescence

Chlorophyll *a* fluorescence transients were measured at room temperature with a Handy Plant Efficiency Analyzer (Hansatech, UK). Illumination was provided by an array of six high intensity LEDs (with a peak of 650 nm) which were focused on the sample surface to provide homogeneous illumination over the exposed area of a sample with 4 mm diameter. Measurements were carried out on leaves dark adapted for 30 min to ensure an initial photochemical activity of zero. During light illumination, chlorophyll *a* fluorescence intensity in dark-adapted leaves rose rapidly from an initial minimal level, F_o_ (the O step), to the maximal level, Fm (P step). Two intermediate steps designated J and I appeared at 2 and 30 ms, respectively; hence, a fast rise of the chlorophyll *a* fluorescence, transient with the notation O–J–I–P, was obtained.

Chlorophyll *a* fluorescence transients were analyzed by utilizing the original data from polyphasic fluorescence transients according to the JIP test [33,53,54]. The following fluorescence parameters were calculated using the JIP test:

The maximum quantum yield of photosystem II (F_v_/F_m_), F_v_/F_m_ = (F_m_-F_o_)/F_m_; the probability that a trapped exciton moves an electron into the electron transport chain beyond Q_A_^−^ (Ψ_o_), Ψ_o_ =1-V_j_ = 1-(F_2ms_-F_o_)/(F_m_-F_o_); the normalized relative variable fluorescence at the K band (W_k_, K indicates fluorescence extensity at 0.3 ms), W_k_ = (F_0.3 ms_ − F_o_)/(F_2 ms_ − F_o_).

### Measurements of chlorophyll fluorescence

Modulated chlorophyll fluorescence was measured with an FMS-2 pulse-modulated fluorometer (Hansatech, UK). The light-fluorescence measurement protocol was as follows: the light-adapted leaves were continuously illuminated by actinic light at 800 μmol m^−2^ s^−1^ from the FMS-2 light source, steady-state fluorescence (F_s_) was recorded after a 2 min illumination, and 0.8 s of saturating light of 8000 μmol m^−2^ s^−1^ was imposed to obtain maximum fluorescence in the light-adapted state (F_m_’). The actinic light was then turned off, and the minimum fluorescence in the light-adapted state (F_o_’) was determined by a 3 s illumination with far-red light.

The following parameters were then calculated [55]:

1 $$ \mathrm{Electron}\ \mathrm{transport}\ \mathrm{rate},\mathrm{E}\mathrm{T}\mathrm{R}=\Phi \mathrm{P}\mathrm{SII}\times \mathrm{P}\mathrm{F}\mathrm{D}\times 0.5\times 0.84 $$

2 $$ \mathrm{Photochemical}\ \mathrm{quenching},\mathrm{q}\mathrm{P}=\left({\mathrm{F}}_{\mathrm{m}}^{\hbox{'}}\hbox{-} {\mathrm{F}}_{\mathrm{s}}\right)/\left({\mathrm{F}}_{\mathrm{m}}^{\hbox{'}}\hbox{-} {\mathrm{F}}_{\mathrm{o}}^{\hbox{'}}\right) $$

3 $$ \mathrm{N}\mathrm{o}\mathrm{n}\hbox{-} \mathrm{photochemical}\ \mathrm{quenching},\mathrm{N}\mathrm{P}\mathrm{Q}=\left({\mathrm{F}}_{\mathrm{m}}\hbox{-} {\mathrm{F}}_{\mathrm{m}}^{\hbox{'}}\right)/{\mathrm{F}}_{\mathrm{m}}^{\hbox{'}} $$

### Histochemical detection of H_2_O_2_

*In situ* hydrogen peroxide (H_2_O_2_) was detected by DAB staining as previously described [56]. H_2_O_2_ reacts with DAB to form a reddish-brown stain. Treated leaf disks were incubated in DAB solution, pH 5.5, at 1 mg/ml. After incubation in the dark at room temperature for 20 h, samples were boiled in alcohol (96%) for 10 min. After cooling, the leaf discs were extracted at room temperature with fresh ethanol and photographed.

### Measurements of gas exchange

Net photosynthetic rate (Pn), substomatal CO_2_ concentration (Ci) was measured at room temperature (25°C) and 60% relative humidity with a portable system (CIRAS-2, PP Systems, UK). The light intensity was set to 800 μmol m^−2^ s^−1^. CO_2_ concentration were changed every 3 min in a sequence of 2 000, 1 600, 1 200, 800, 600, 400, 300, 200, 150, 100, and 0 μmol mol^−1^. Irradiance and CO_2_ concentration were controlled by the automatic control function of the CIRAS-2 photosynthetic system. Carboxylation efficiency was calculated according the initial slop of Pn-Ci response curve.

### Measurements of soluble sugar and starch in tobacco leaves

The samples (25 leave discs) were grounded in double-distilled water and filtered. The residue was again grounded and filtered. Filtrates were pooled and centrifuged at 10,000 × g for 15 min. The sample solution (0.1 mL) was taken in a test tube and made to 1 mL with double-distilled water. Four millilitre of 0.2% anthrone reagent (0.2 g dissolved in 100 mL conc. H_2_SO_4_) was added, and the contents were heated in a boiling water bath and subsequently cooled. The absorbance was read at 620 nm [57]. A mixture of 1 mL distilled water and 4 mL of 0.2% anthrone served as blank. The final residue of the leaves after filtered was resuspended in 1.6 M perchloric acid and incubated in a water bath at 70°C for 2 h. Then samples were centrifuged at 10000 g for 10 min and the carbohydrated concentration in the supernatant was determined via the anthrone method as described above.

### Measurements of PSI activity and cylic electron flow around PSI

The modulated reflection signal measured at 820 nm (MR_820nm_) provides information about oxidation of PSI (concluding PC and P700). MR_820nm_ were recorded using a Multifunctional Plant Efficiency Analyzer, M-PEA (Hansatech Instrument *Ltd.,* UK).The induction curve of MR_820nm_ of the leaves obtained by saturating red light shows a fast oxidation phase and a following reduction phase. The initial slope of oxidation phase of MR_820nm_ at the beginning of the saturated red light indicates the capability of P700 to get oxidized, which is used to reflect the activity of PSI [17,18,58].

Cylic electron flow around PSI of leaves were measured after leaves were dark adapted for 15 min. After illuminated by far-red light for 20 s, the far-red light was turned off and the MR_820nm_ of the leaves was recorded. The initial increase rate of the MR_820nm_ indicates the intensity of cyclic electron flow around PSI [19]. Statistical analysis.

LSD (least significant difference) was used to analyse differences between different treatments by using SPSS 16.

## Additional files

Additional file 1: Figure S1. The effect of K_2_C_2_O_4_ and KCl treatment on the content of pigment in tobacco leaves at the end of treatment. The “Dark” were dark adapted leaves without any reagent treatment. Different letters indicate significant differences between leaves with different treatments (P < 0.05). Values shown are means ± SE (n = 5). Additional file 2: Figure S2. Effect of HCl (pH 4.0), H_3_PO_4_ (pH 4.0), H_2_C_2_O_4_ (40 mM, pH adjusted to 4.0), K_2_C_2_O_4_ (40 mM) and KCl (80 mM) treatment on Wk **(A)** and K band (O-J normalized, **B)** in tobacco leaves. Leaf discs (10 mm diameter) were infiltrated with HCL (pH 4.0), H_3_PO_4_ (pH 4.0), H_2_C_2_O_4_ (40 mM, pH adjusted to 4.0), K_2_C_2_O_4_ (40 mM) and KCl (80 mM) under darkness for 3 h, followed by exposure to intense light (800 μmol m^−2^ s^−1^) for 2 hours. CK were leaves without any reagent treatment. Different letters indicate significant differences between leaves with different treatments (P < 0.05). Values were means ± SE (n = 8). Additional file 3: Figure S3. Effect of HCl (pH 4.0), H_3_PO_4_ (pH 4.0), H_2_C_2_O_4_ (40 mM, pH adjusted to 4.0), K_2_C_2_O_4_ (40 mM) and KCl (80 mM) treatment on Ψ_o_ **(A)** and OJIP curves (O-P normalized, **B)** in tobacco leaves. Leaf discs (10 mm diameter) were infiltrated with HCL (pH 4.0), H_3_PO_4_ (pH 4.0), H_2_C_2_O_4_ (40 mM, pH adjusted to 4.0), K_2_C_2_O_4_ (40 mM) and KCl (80 mM) under darkness for 3 h, followed by exposure to intense light (800 μmol m^−2^ s^−1^) for 2 hours. CK were leaves without any reagent treatment. Different letters indicate significant differences between leaves with different treatments (P < 0.05). Values were means ± SE (n = 8). Additional file 4: Figure S4. The Pn-CO_2_ curves of tobacco leaves treated with different concentrations (0, 20, 40, 60 mM) of K_2_C_2_O_4_ and 120 mM KCl. The petioles of detached leaves were dipped into treatment solutions before measurement in the dark for 3 hours. CK were leaves without K_2_C_2_O_4_ and KCl treatment. Additional file 5: Figure S5. The effect of 40 mM K_2_C_2_O_4_ and 80 mM KCl treatment on the F_o_ in tobacco leaves treated with high-light for 2 hours. CK were leaves without K_2_C_2_O_4_ and KCl treatment. Different letters indicate significant differences between leaves with different treatments (P < 0.05). Values were means ± SE (n = 8).

## Abbreviations

Am: Net photosynthesis rate in saturated CO_2_
CE: Carboxylation efficiency
CM: Chloramphenicol
DAB: 3, 3-diaminobenzidine
F_v_/F_m_: Maximal quantum yield of PSII
H_2_O_2_: Hydrogen peroxide
Pn: Net photosynthesis rate
PSI: Photosystem I
PSII: Photosystem II
RC/CSm: Density of Q_A_-reducing PSII reaction centres
ROS: Reactive oxygen species
RuBP: Ribulose-1,5-bisphosphate
IAM: Iodoacetamide
S. sclerotiorum: Sclerotinia sclerotiorum (Lib.) de Bary
Wk: Normalized relative variable fluorescence at the K step
π_o_: Exciton efficiency of electron transport beyond Q_A_
