# Supplementary figures and images for: Mechanisms by which the infection of Sclerotinia sclerotiorum (Lib.) de Bary affects the photosynthetic performance in tobacco leaves
Source: BMC Plant Biol. 2014 Sep 23;14:240. doi: 10.1186/s12870-014-0240-4 (PMC4180539; doi:10.1186/s12870-014-0240-4)

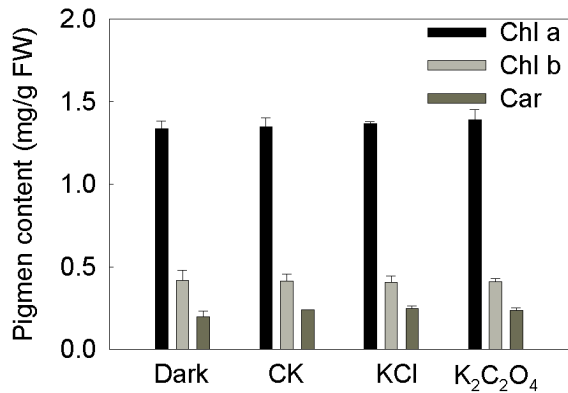

Supplement: Additional file 1: Figure S1. — The effect of K2C2O4 and KCl treatment on the content of pigment in tobacco leaves at the end of treatment. The “Dark” were dark adapted leaves without any reagent treatment. Different letters indicate significant differences between leaves with different treatments (P < 0.05). Values shown are means ± SE (n = 5). [file 12870_2014_240_MOESM1_ESM.pdf]

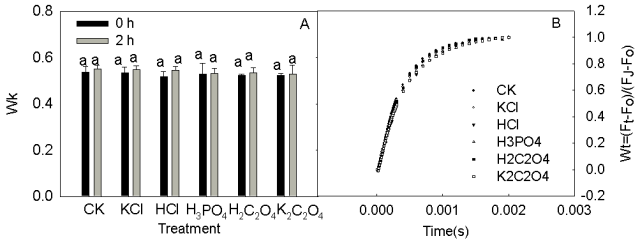

Supplement: Additional file 2: Figure S2. — Effect of HCl (pH 4.0), H3PO4 (pH 4.0), H2C2O4 (40 mM, pH adjusted to 4.0), K2C2O4 (40 mM) and KCl (80 mM) treatment on Wk (A) and K band (O-J normalized, B) in tobacco leaves. Leaf discs (10 mm diameter) were infiltrated with HCL (pH 4.0), H3PO4 (pH 4.0), H2C2O4 (40 mM, pH adjusted to 4.0), K2C2O4 (40 mM) and KCl (80 mM) under darkness for 3 h, followed by exposure to intense light (800 μmol m−2 s−1) for 2 hours. CK were leaves without any reagent treatment. Different letters indicate significant differences between leaves with different treatments (P < 0.05). Values were means ± SE (n = 8). [file 12870_2014_240_MOESM2_ESM.pdf]

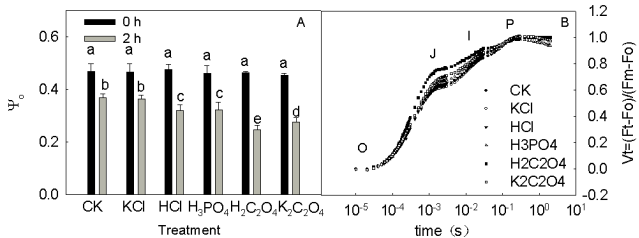

Supplement: Additional file 3: Figure S3. — Effect of HCl (pH 4.0), H3PO4 (pH 4.0), H2C2O4 (40 mM, pH adjusted to 4.0), K2C2O4 (40 mM) and KCl (80 mM) treatment on Ψo (A) and OJIP curves (O-P normalized, B) in tobacco leaves. Leaf discs (10 mm diameter) were infiltrated with HCL (pH 4.0), H3PO4 (pH 4.0), H2C2O4 (40 mM, pH adjusted to 4.0), K2C2O4 (40 mM) and KCl (80 mM) under darkness for 3 h, followed by exposure to intense light (800 μmol m−2 s−1) for 2 hours. CK were leaves without any reagent treatment. Different letters indicate significant differences between leaves with different treatments (P < 0.05). Values were means ± SE (n = 8). [file 12870_2014_240_MOESM3_ESM.pdf]

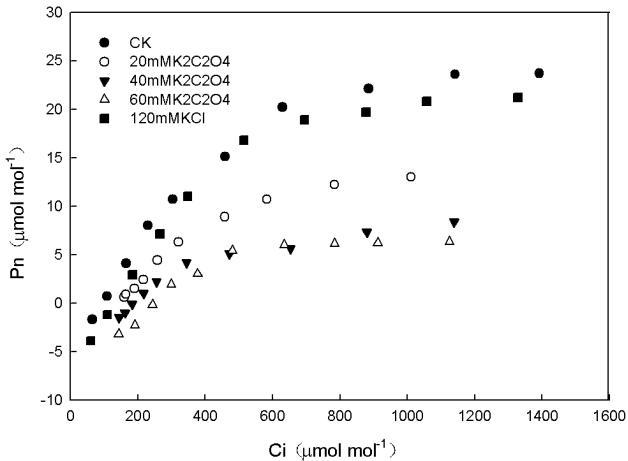

Supplement: Additional file 4: Figure S4. — The Pn-CO2 curves of tobacco leaves treated with different concentrations (0, 20, 40, 60 mM) of K2C2O4 and 120 mM KCl. The petioles of detached leaves were dipped into treatment solutions before measurement in the dark for 3 hours. CK were leaves without K2C2O4 and KCl treatment. [file 12870_2014_240_MOESM4_ESM.pdf]

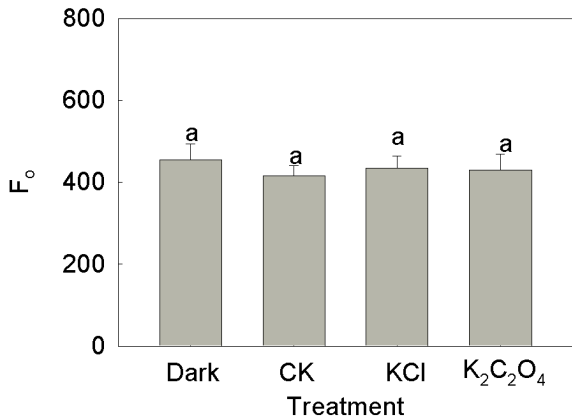

Supplement: Additional file 5: Figure S5. — The effect of 40 mM K2C2O4 and 80 mM KCl treatment on the Fo in tobacco leaves treated with high-light for 2 hours. CK were leaves without K2C2O4 and KCl treatment. Different letters indicate significant differences between leaves with different treatments (P < 0.05). Values were means ± SE (n = 8). [file 12870_2014_240_MOESM5_ESM.pdf]
